# Comparative analysis of IgG responses to *Plasmodium falciparum* MSP1p19 and PF13-DBL1α1 using ELISA and a magnetic bead-based duplex assay (MAGPIX®-Luminex) in a Senegalese meso-endemic community

**DOI:** 10.1186/1475-2875-13-410

**Published:** 2014-10-17

**Authors:** Ronald Perraut, Vincent Richard, Marie-Louise Varela, Jean-François Trape, Micheline Guillotte, Adama Tall, Aissatou Toure, Cheikh Sokhna, Inès Vigan-Womas, Odile Mercereau-Puijalon

**Affiliations:** Institut Pasteur de Dakar, Unité d’Immunologie, Dakar, Sénégal; Institut Pasteur de Dakar, Unité d’Epidémiologie, Dakar, Sénégal; Institut de Recherche pour le Développement (IRD), URMITE, Dakar, Sénégal; Institut Pasteur, Unité d’Immunologie Moléculaire des Parasites, 25-28 Rue du Dr Roux, 75015 Paris, France; Institut Pasteur de Madagascar, Unité d’Immunologie, Antananarivo, Madagascar

**Keywords:** Malaria, *Plasmodium falciparum*, ELISA, IgG, Surface antigens, MSP1p19, PfEMP1-PF13, Multiplex, MAGPIX, Senegal, Ndiop

## Abstract

**Background:**

Numerous *Plasmodium falciparum* antigens elicit humoral responses in humans living in endemic areas. Use of multiplex assays is a convenient approach to monitor the antibody response against multiple antigens, but to integrate multiplex assay-derived data with datasets, generated previously using ELISA, comparative studies are needed. This work compares antibody responses to two *P. falciparum* antigens monitored using both technologies.

**Methods:**

The IgG response against the merozoite surface protein-1 PfMSP1p19 and the PF13-DBL1α1 domain of the *P. falciparum* Erythrocyte Membrane Protein1, expressed by the rosette-forming parasite 3D7/PF13 (PF13), was investigated using ELISA and a MAGPIX®-Luminex duplex assay. Archived plasma samples collected before the rainy season from 217 villagers living in Ndiop, a Senegalese meso-endemic setting, were studied. ROC analysis was used to define the optimal antibody measure readout. Association of antibody levels with protection against clinical malaria was analysed using Poisson regression in a retrospective study from active case detection records performed during the 5.5-month transmission season that followed blood sampling.

**Results:**

There was a strong positive correlation (*P* <10^-3^) between ELISA and MAGPIX®-Luminex-MFI (median fluorescence intensity) values for antibody to PfMSP1p19 (rho = 0.78) and PF13-DBL1α1 (rho = 0.89), with a similar degree of concordance in all age groups. Antibody levels to both antigens were high but displayed a different age-associated pattern. Independent age-adjusted Poisson regression analysis showed a significant association with protection only for IgG responses to MSP1p19 (*P* <0.01 RR = 0.71 [0.53-0.93]) measured by ELISA.

**Conclusion:**

The individual ELISA and duplex-MAGPIX assays provide a concordant evaluation of age-associated antibody responses to MSP1p19 and PF13-DBL1α1, irrespective of the formulation of antibody levels (values, ratios or ROC-adjusted figures) but do diverge with regard to the association of antibody levels with clinical protection in age-adjusted models. This may reflect incomplete overlap of the epitopes presented in the two formats. Further development for multiplex assessment of antibody responses to a larger panel of antigens with the robust and cost effective MAGPIX®-Luminex technology is warranted.

**Electronic supplementary material:**

The online version of this article (doi:10.1186/1475-2875-13-410) contains supplementary material, which is available to authorized users.

## Background

Malaria is a major threat in tropical and subtropical regions, with nearly 50% of the world population exposed to infective bites by anopheles mosquitoes [1]. Malaria parasites expose a large array of antigens both at the pre-erythrocytic and erythrocytic stages. Although it has been clearly demonstrated that antibodies play a crucial role in immune protection to malaria [2, 3], the precise antigenic targets and mechanism of protective immunity remain uncertain [4]. Numerous studies have attempted to use the antibody response to specific parasite antigens to evaluate exposure to malaria in both immune and naive populations. Responses to some antigens were shown to partially correlate with clinical protection in immune or vaccinated populations [5], but no consensus has emerged yet.

Multiple studies [6, 7] investigated antibody responses in endemic populations using the enzyme-linked immunosorbent assay (ELISA). This technique requires separate, independent testing for each individual antigen, which is labour intensive and differs from the *in vivo* situation with multiple parasite antigens exposed simultaneaously. Moreover, the increasing number of newly discovered antigens [8] calls for implementation of multi-target detection capacity either for analysing the immune signature of infection or for pre- or post-vaccination investigations [9]. Furthermore, often only small quantities of blood are ethically available for analysis in malaria-endemic populations, including neonates or finger-prick samples from adults. Therefore, a reliable assay that measures antibody responses simultaneously to several antigens with a few microlitres of blood would be highly advantageous.

Multiplex immune detection assays, which measure multiple antigens simultaneously, can be performed in several formats using micro-arrays [10–12] or Luminex™ (Luminex Corp, Austin, USA) xMAP fluorescent-coded beads, where each individual antigen is covalently linked to specific colour-coded microspheres such that the reading device can classify each bead set separately. Luminex™ instrumentation generally quantifies laser-induced fluorescent signals from each bead using flow cytometric technology. This methodology has recently been used for multiplex detection of malaria antigens [13–16]. A convenient recently developed alternative is the MAGPIX® technology (Millipore, MA, USA), which uses colour-coded magnetic beads displayed in a monolayer, which are detected with a *light-emitting diode (*LED) instead of laser system and imaged using a *charge-coupled device (*CCD) camera. This platform considerably reduces the costs of the multiplex approach since antigen is immobilized on a much smaller surface area compared to microplate wells, and sample volumes are reduced compared to ELISA. However, there are important differences between bead-based assays and ELISA. The beads bind antigen via covalent cross-linking of protein lysine residues to bead carboxylic acids using a carbodiimide compound, as opposed to ELISA where antigen coating of the well surface results from hydrophobic interactions with the plastic plate. Thus, different protein residues interact with the supports in the two methodologies, possibly modifying the exposure of antigen epitopes.

The present study was designed to compare the MAGPIX®-Luminex and ELISA approaches by measuring antibody responses to two recombinant *Plasmodium falciparum* antigens, the baculovirus-expressed PfMSP1p19 derived from merozoite surface protein 1 [17], and PF13, the NTS-DBL1α _domain of *P. falciparum* Erythrocyte Membrane antigen 1 of the PF13_0003 antigen expressed by the rosette-forming 3D7/PF13 clone [18]. A retrospective analysis was conducted on blood samples collected before the rainy season from individuals living in the village of Ndiop, a meso-endemic setting located in southern Senegal. Prevalence and levels of antibody response were assessed using both methodologies and the relationship of antibody responses with morbidity, as measured by active case surveillance during the following 5.5 months, was explored.

## Methods

### Study area, study design, ethic statements and procedures

The study was carried out in Ndiop, a Senegalese village with seasonal transmission where a long-term longitudinal survey designed to study acquisition and maintenance of natural immunity has been carried out over more than 20 years [19–22]. The project protocol and objectives were carefully explained to the assembled villagers, and informed consent was obtained from all participants or their parents or guardians on a voluntary consent form written in both French and in Wolof, the local language. The protocol was approved by the Senegalese National Health Research Ethics Committee. An agreement between Fondation Institut Pasteur de Dakar, Institut de Recherche pour le Développement (IRD) and the Ministère de la Santé et de la Prévention of Senegal defines all research activities in Ndiop. The project is re-examined annually by the Conseil de Fondation de l’Institut Pasteur de Dakar and approved by the assembled village population. Informed consent is individually renewed from all subjects; anyone can withdraw from the study and the follow-up procedure at any time.

Active clinical surveillance was carried out during the 5.5-month transmission period of year 2002, such that each villager was monitored daily at home and blood films were analysed in case of fever [20, 21]. The protocol included recording all febrile episodes and controlled use of anti-malarial drugs by the medical staff. A malaria attack was defined as an association of symptoms indicative of malaria with parasitaemia >30 trophozoites/100 leukocytes. Anti-malarial drugs were administered under supervision of the medical staff following diagnosis of clinical malaria. Malaria attacks were considered independent if separated by more than 15 days.

The cumulative entomological inoculation rate (EIR) was monitored as described [23]. The estimated EIR for the year 2002 transmission season was 17.9 infective bites/individual from the beginning of September to the end of November, with no detectable transmission in July, August and December 2002.

### Antigens

The procedure for protein expression and purification of the recombinant PF13 (amino acids 1–486 of PF13_0003, with all predicted N-glycosylation sites mutated to NxA and a C-terminal hexa-His tag) has been described [24]. Soluble recombinant protein corresponding to *P. falciparum* MSP1p19 was produced in the baculovirus/insect cell expression system and purified by metallo-affinity chromatography as described [17].

### ELISA

PF13 and PfMSP1p19 were coated on Immulon-4 plates (Dynatech) at 1 and 0.5 μg.mL^-1^, respectively. Sera were used at a 1/200 dilution. All other procedures were as described [24, 25]. Each plate included two positive controls: a pool of human Immune IgG (kind gift from Prof M Hommel) and a pool of 30 sera collected in 2000–2 from immune adults from Dielmo, a holoendemic village located 5 km apart from Ndiop [21, 22]. The negative naïve control was a pool of non-immune sera from blood donors living in France. For inter-assay comparisons, results were expressed as absorbance ratios corresponding to OD-sample/OD-naïve. Positive responders were individuals with an absorbance ratio over 2 corresponding to the mean OD of naïve controls +2SD.

### Coupling of antigens to beads

The covalent coupling of PF13 and PfMSP1p19 to carboxylated magnetic Luminex microspheres by the carbodiimide reaction (Luminex Corp, Austin, USA) was done using the xMAP® Antibody Coupling Kit (ref 40–50016, Luminex Corp, Austin, USA) according to the manufacturer’s instructions. Briefly, 2.5×10^6^ beads from colour regions 12 and 13 were resuspended after sonication and rotative mixing in microcentrifuge reaction vials and washed twice before adding the activation buffer. The working volume was 500 μL; all washing steps or buffer changing were done after 1–2 min centrifugation at 8,000 × g and 1-min magnetic pelletting using the Luminex® Magnetic plate separator (Luminex Corp, Austin, USA), followed by 30-sec vortexing and 30-sec sonication in water-bath sonicator to optimally disperse the beads. Ten μL carbodiimide hypochloride (EDC) was then added and incubated 20 min with a vortexing step after 10 min. After three washes, 5 μg antigen per million beads (amount determined from prior optimisation procedure, manuscript submitted) was added in the activation buffer in a final volume of 500 μL and kept under rotation mixing (15–30 rpm) in the dark for two hours. After three steps of pelletting and washing, the supernatant was removed and replaced by 1 mL wash buffer and kept in the dark at 2-8°C after another vortexing and sonication step to disperse the microspheres. The final count of beads using cell counter showed 96% recovery (2.4×10^6^ beads). Efficient coupling of antigen was controlled using mouse anti-His tag antibody. The coupled microspheres were kept in the washing/storage buffer at 4°C in the dark until use.

### Bead-based assay for IgG antibodies to *PF13 and PfMSP1p19*

The custom magnetic bead-based MAGPIX®-Luminex assay (MBA), performed in a dimly lit room, was adapted to parallel the working steps used in the standard ELISA technique. The duplex mixture of PF13 and PfMSP1 microspheres was prepared and kept in an opaque vial; 2.5 μL aliquots containing 3,000 beads per antigen were dispensed to individual wells of a white, polystyrene, opaque, round-bottomed microtitre plate (Ref. 103977741, Fisher Scientific, Illkirch, France); 100 μL plasma diluted 1/200 in PBS Tween 0.01% BSA 1% (PBSB) was added in duplicate wells, mixed and incubated with the beads protected from light on a microplate shaker (IKA®MTS, Wilmington, NC, USA) at 350 rpm for 45 min. Removal of plasma and two washing steps of magnetic-pelletted beads with 100 μL PBSB were done, 100 μL phycoerythrin-labelled goat anti-human IgG diluted 1:500 (gamma- chain specific, F(ab’)_2_ fragment-R-phycoerythrin (P-8047, Sigma, St. Louis, MO, USA) in PBSB was then added to each well and incubated in the dark, with shaking at 350 rpm for 45 min. This step was followed by two careful washes of the plates as above, with 100 μL/well of PBSB. The beads were then resuspended in 120 μL PBSB and analysed on a Multiplex MAGPIX system (Millipore, MA, USA) using the xPONENT 4.1 software for acquisition and assay design. The reader was set to read a minimum of 50 beads of unique fluorescent signature per region with the output of median fluorescence intensity (MFI) per sample as stated by manufacturer’s instructions. Antibody response was considered positive for MFI values if the signal was greater than twice the background signal [mean of 6 determinations +2 SD of the negative pool of non-immune control sera].

The MFI ratio was calculated as MFI sample/MFI naïve (MFI naïve being the mean MFI signal +2 SD of naïve controls), and as for ELISA, responders were those with MFI ratio > 2. The prevalence of positive antibody responses was also calculated using receiver operating characteristic (ROC) curve procedure (see below).

### Statistical analysis

Comparisons for categorical variables were done using the Fisher exact test, continuous variables of antibody responses were analysed using the Kruskal Wallis and the Spearman rank correlation test for non-normally distributed data. The ROC (Receiver Operating Characteristic) curve analysis was used to determine the best threshold point for optimal positivity according to the two methods. P values <0.05 were considered significant.

A Poisson regression model was used to analyse the relationship between antibody responses and the incidence of malaria attacks during the follow-up period. Antibody responses to MSP1 and PF13, expressed as OD or MFI, OD or MFI ratios or categorized into responders *vs.* non-responders, were analysed as monoplex and duplex responses. For analysis, the age stratification was based on the age-dependent distribution of the parasite and clinical data determined for this site [20]. Malaria attacks were considered independent if separated by more than 15 days. For each villager, the follow-up time was calculated as the number of days actually spent in the village during the 5.5 months of follow-up. Villagers who were away from the village for more than 30 days during the follow-up period were excluded from the malaria incidence analysis. Follow-up time was also adjusted for individuals who received anti-malarial treatment, by excluding a period of eight to 15 days after the first day of treatment (eight days for quinine, ten days for chloroquine and 15 days for sulphadoxine-pyrimethamine treatments, respectively). Statistical analyses were performed with Egret 3.01®, R and Statview 5.0® softwares.

## Results

### Characteristic of the cohort

Cross-sectional blood sampling was done for 217 healthy villagers aged 3.4 to 76.9 years. Five age classes were defined for analysis, i.e., <five years, five to nine years, ten to 14 years, 15–29 years, and >30 years. Detailed characteristics of the cohort are summarized in Table 1. At the time of sampling in July, 19% of the villagers had a microscopically positive blood smear. Individuals recruited for the study had not used anti-malarial medication for at least four weeks prior to blood sampling.

**Table 1:** **Characteristics of the cohort from Ndiop analysed in this study**

	Age groups (years)					
	<5	5-9	10-14	15-29	≥30	Overall
Number of villagers	11	39	34	65	68	217
Sex ratio F/M	5/6	19/20	17/17	39/26	36/32	116/101
Median age (years)	3.9	7.3	12.4	20.1	43.9	22.8
No Indiv. Hb AA/AS/AC	8/3/0	33/3/3	32/1/1	55/10/0	54/13/1	182/30/5
% parasitemic individuals*	9%	21%	38%	16%	11%	19%
Overall number of clinical attacks during follow up (%)	24 (10%)	108 (47%)	51 (22%)	33 (16%)	13 (6%)	229 (100%)

*Individuals with positive blood smear on the day of blood withdrawal.

### ELISA and magnetic-beads MAGPIX®-Luminex assay (MBA)

ELISA and MBA were done independently on each blood sample. MBA were performed during three consecutive days within one week following antigen coupling. MBA showed excellent specificity with very low MFI results on multiple naïve or negative individuals (MFI from 85 to 120, with mean values of 95, 105 for MSP1p19 and PF13, respectively). Efficient coupling of antigen was controlled using mouse anti-His tag antibody (MFI above 10,000 for each region) and high MFI signal were observed when using mouse monospecific control antibodies (MFI above 8,000).

For the analysis of antibody responses using the two techniques, several read out formulations were used for antibody levels, namely observed OD and MFI values as well as calculated OD- or MFI-ratio, as values and/or ratios are commonly used in seroepidemiology. Their different dynamic range provide a different appreciation of the data. These two types of formulations were used to analyse response levels by age group and their association against subsequent clinical malaria in order to identify the best read out to use in further studies. Seroprevalence was calculated from OD- or MFI-ratio or ROC adjusted results.

### General concordance between duplex MBA and monoplex ELISA estimates of antibody responses

**Figure 1:**
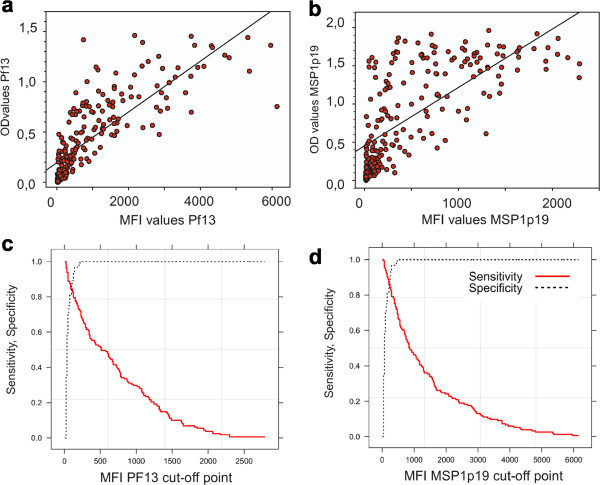
**Correlation between the results from ELISA and duplex MBA using the Spearman’s rank correlation test and ROC curves calculation output.** ELISA OD (Y axis) *vs* MFI (X axis) values are plotted for IgG responses against PF13 **(a)** and MSP1p19 **(b)**. The regression line indicates a significant correlation (*P* <10^-3^) of antibody responses measured by two methods for PF13 (rho = 0.89) and for MSP1p19 (rho = 0.78). The ROC curves plotting specificity *vs* sensitivity of the two methods, used to calculate the best cut-off point, are shown for PF13 **(c)** and MSP1p19 **(d)**.

As illustrated in Figure 1, there was a significant (*P* <10^-3^) positive correlation between MFI and OD values for antibody responses to PF13 (Figure 1a, rho = 0.89) and to MSP1p19 (Figure 1b, rho = 0.78) monitored by the two methodologies. When including only positive responders (*ie*, individuals with OD-ratio >2), the correlation remained significantly positive (*P* < 10^-3^), rho = 0.80 and 0.65 for PF13 and MSP1p19, respectively. When stratifying by age group, antibody levels showed a similar degree of concordance of the two techniques in all age groups (<5, 5–10, 10–15, 15–29, and ≥30 years) for PF13 (rho = 0.89, 0.90, 0.83, 0.89, 0.85, respectively) and MSP1p19 (rho = 0.97, 0.68, 0.82, 0.80, 0.72, respectively).

The ROC curves analysis was used to determine cut-off points of optimal sensitivity, specificity of MFI *vs* ELISA. Plotting of the curve is shown in Figure 1c for PF13 with a cut-off at MFI = 214 (sensitivity = 86%, specificity = 87%) and in Figure 1d for MSP1p19 where cut-off was at MFI = 114 (sensitivity = 83%, specificity =77%).

### Prevalence of antibody responses against PF13 and MSP1p19

A high prevalence of IgG responses against both antigens was observed both using ELISA and MBA as well using the ROC-defined thresholds (Table 2). Seroprevalence to PF13 measured by the two techniques did not differ significantly, 71 *vs* 65% of responders at the village level, by ELISA and MBA, respectively. Using the ROC-corrected calculation, seroprevalence was not modified compared to MFI ratios output in all age groups. Seroprevalence to PF13 determined by ELISA was low in children under five years, it increased quite significantly in the five to nine years group , further increased reaching 94% in the ten to 14 years group, and decreased in older individuals (Fisher exact test, *P* < 0.01). Seroprevalence calculated from MFI-ratios and ROC-adjusted values was lower than estimated from ELISA data. The age distribution also showed a sharp increase during childhood. Compared to ELISA, there was a smaller, non-significant increase in the ten to 14 year-old children, and values plateaued thereafter.

**Table 2:** **Prevalence of IgG antibody responses against MSP1p19 and PF13 according to different formulations of ELISA and duplex MBA data**

Villagers		Antibody responses to PF13					Antibody responses to PfMSP1p19				
Age-class	No	OD-ratio*	%resp.	MFI-ratio*	%resp.	%ROC**	OD-ratio	%resp.	MFI-ratio	%resp.	%ROC
<5y	11	1.9	**27%**	2.3	**18%**	**18%**	3.7	**64%**	3.4	**36%**	**36%**
5-9y	39	6.0	**51%**	8.7	**59%**	**54%**	3.6	**49%**	3.7	**33%**	**56%**
10-14y	34	10.3	**94%**	12.5	**71%**	**71%**	5.1	**74%**	5.9	**59%**	**74%**
15-29y	65	9.4	**74%**	11.1	**68%**	**68%**	6.6	**80%**	6.5	**57%**	**63%**
≥30y	68	8.7	**74%**	8.6	**72%**	**72%**	7.5	**85%**	6.9	**69%**	**79%**
all	217	8.3	**71%**	9.7	**65%**	**65%**	5.9	**74%**	5.8	**56%**	**67%**

*Mean values of antibody responses measured with ELISA and duplex magpix test.

**Incidence of responders using optimal threshold defined by ROC analysis.

Seroprevalence to MSP1p19 measured by ELISA was also high, reaching 74% at the village level by ELISA and estimated at 56 or 67% by MFI-ratios or ROC-adjusted MFI, respectively. Prevalence calculated from MFI-ratios was consistently lower in all age classes compared to ELISA OD-ratios and ROC-adjusted MFI (P <0.01). When considering the MFI optimal threshold defined by ROC analysis, 25 MFI ratio-negative individuals were classified as positive and the percentage of responders increased to intermediate values between those calculated from ELISA OD-ratios and MFI-ratios. As shown in Table 2, seroprevalence estimated from ELISA OD-ratios was substantial in the < five years age group and increased in the > nine years old children, and adults. Intriguingly, seroprevalence tended to be lowest in the five to nine years old children, this difference being significant compared to the 15–29 or ≥30 years age groups (Fisher exact test, *P* < 0.01). Such an age-associated profile was not observed for antibodies monitored using MBA.

### Comparison of age distribution of antibody levels estimated using ELISA and MBA

For the two antigens, there was a low but significant relationship between age and antibody responses measured by ELISA and MBA (Spearman rank test, Rho = 0.17 to 0.21, *P* <0.05).

**Figure 2:**
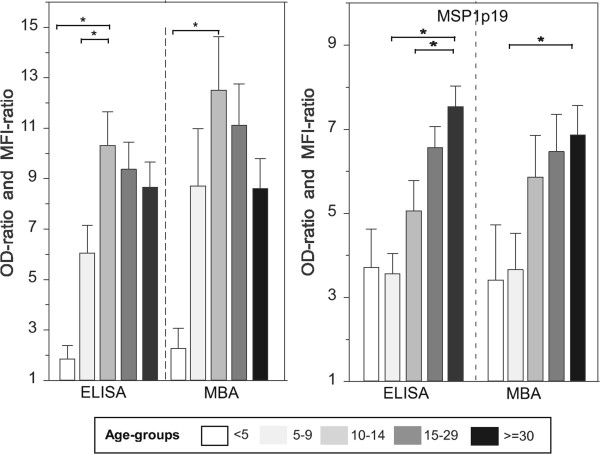
**Level of IgG responses to PF13 and PfMSP1p19 measured by individual ELISA and duplex MBA.** OD- and MFI-ratios of IgG responses to PF13 and MSP1p19 are plotted as histograms (mean + SE). Antibody responses were stratified according to five age groups (<5, 5–9, 10–14, 15–29 and >30 years; symbols used range from empty, pale grey, light grey, dark grey and black, respectively). Brackets and asterisk indicate significant differences (*P* <0.05) between levels of antibody responses in different age groups.

The age-stratified profiles of mean IgG responses to both antigens are shown on Figure 2. Very similar age-stratified profiles were observed using the two methodologies, and they were antigen-specific. There was a sharp increase of antibody levels to PF13 after the age of five years, peaking significantly in the ten to 14 year old children, (Mann–Whitney rank test, *P* <0.01), and slightly decreasing in the older age group. For MSP1p19 Ag, antibody levels were moderate in the two younger age groups (<5 and 5–9 y) and continuously increased with age. The difference was significant between the younger age group and the oldest one in all cases. Importantly, the age profile was independent of the read out formulation. Figure 2 shows the OD-ratio and MFI-ratio data, age-distribution with OD and MFI values are shown in Additional file 1.

Importantly, there was no significant relationship between the magnitude of antibody responses to PF13 and to MSP1p19 with haemoglobin phenotype of individuals (AA *vs* AS). Moreover, detectable *P. falciparum* asymptomatic carriage, as evidenced by thick blood smear count, had no impact on antibody values.

### Relationship between antibody responses and occurrence of clinical attacks

**Figure 3:**
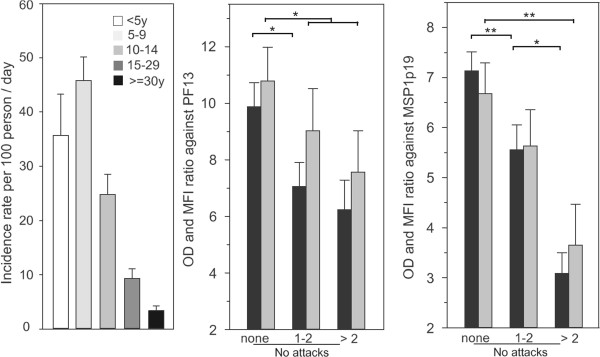
**Incidence of clinical malaria attacks during the 5.5-month follow-up and its relationship to levels of antibody responses against PF13 and MSP1p19.** The left panel shows the mean incidence of malaria attacks by age group. The middle and right panels show OD-ratios (black) and MFI-ratios (grey) values of antibody responses to PF13 or MSP1p19 plotted as histograms (+SE) by occurrence of clinical attacks in the follow-up period stratified as 0 *vs* 1–2 *vs* >2 confirmed clinical malaria attacks. The number of individuals in each stratified group was 107 (mean age 32.5 [3.9-76.9]), 76 (mean age 19.4 [3.7-74.6]), 34 (mean age 8.7 [3.4-16.1]) with 0 *vs* 1–2 *vs* >2 clinical attacks, respectively. Brackets and asterisk indicate significant differences (*P* < 0.05) between antibody responses in different categories.

A total of 217 individuals were considered for longitudinal follow-up period (1 July-31 December); 229 clinical malaria episodes were recorded. The mean incidence rate of malaria attacks (number of malaria attacks per 100 person-months) was 17.7 (95% CI: [14.6-20.9]). It differed between age groups (Kruskal-Wallis test, *P* <0.01), with a higher rate (45.8) in the five to nine years group and lower (3.3) in the ≥30 years age group. The cumulative incidence of clinical malaria attacks is shown in Figure 3, left panel, with 24, 108, 51, 33, and 13 clinical malaria episodes recorded in the < five, five to nine, ten to 14, fifteen to29 and ≥30 years age groups, respectively.

Antibody levels to PF13 and MSP1p19 at recruitment, stratified by number of clinical malaria attacks (0 *vs* 1–2 *vs* >2) experienced during the following transmission season are shown in Figure 3 (central and right panel, respectively). For both antigens, a significantly lower level of antibody response was evidenced in individuals who experienced one or more clinical attacks during the follow-up period compared to those who experienced no clinical malaria episode during that period.

A Poisson regression was used to analyse the relationship between antibody responses and the incidence of clinical malaria episodes during the 5.5-month period of follow-up. The MSP1p19 or PF13 IgG levels expressed as ELISA OD-ratio or MFI-ratio were significantly associated with incidence of malaria attacks in univariate (monoplex antigen) analysis (Table 3). The duplex antigen analysis showed a significant association of malaria attack incidence with IgG responses to MSP1p19 expressed as OD values, OD-ratio or dichotomized into positive *vs* negative ELISA responses (Table 3), but there was no significant relationship with MSP1p19 MFI or MFI-ratio values or positive *vs* negative dichotomized MFI-ratio. In this analysis, antibody levels to PF13 were not associated with incidence of malaria attacks, whatever the antibody-related variable considered. In the age-adjusted analysis used as the final model, where age was categorized in five classes (Table 3), incidence of malaria attack was significantly associated with IgG responses to MSP1p19 dichotomized into positive *vs* negative ELISA responses (*P* = 0.01, RR = 0.71; CI: 95% [0.53-0.93]). Dichotomisation of high *vs* low antibody responses to MSP1p19 (OD-ratio >7) [6, 25] did not show significant association with reduced number of malaria attacks (*P* > 0.05). In this age-adjusted model, antibody levels to PF13 were also unrelated to incidence of malaria attacks.

**Table 3:** **Summary of univariate and multivariate Poisson regression analysis of relationship between PF13 and MSP1 antibody response with incidence rate of malaria attacks in Ndiop**

	Univariate individual antigen analysis			PF13 and MSP1p19 adjusted model			PF13 and MSP1p19 model adjusted for age		
	OR	95% CI	***P***	OR	95% CI	***P***	OR	95% CI	***P***
**ELISA OD**									
PF13	0.70	[0.6-0.9]	<0.01	0.89	[0.74-1.1]	0.25	0.88	[0.7-1.1]	0.19
MSP1	0.46	[0.37-0.59]	<0.01	0.49	[0.38-0.62]	<0.001	0.81	[0.62-1.1]	0.12
**MFI**									
PF13	0.99	[0.99-0.99]	0.02	0.99	[0.99-1.0]	0.12	0.99	[0.99-1.0]	0.06
MSP1	0.99	[0.99-0.99]	<0.01	0.99	[0.99-0.99]	<0.01	0.99	[0.99-1.0]	0.58
**OD ratio**									
PF13	0.96	[0.94-0.98]	<0.01	0.98	[0.96-1.0]	0.06	0.98	[0.96-1.0]	0.08
MSP1	0.88	[0.85-0.92]	<0.01	0.89	[0.86-0.93]	<0.001	0.97	[0.93-1.0]	0.13
**MFI ratio**									
PF13	0.98	[0.97-0.99]	0.02	0.99	[0.9-1.0]	0.11	0.98	[0.97-1.0]	0.06
MSP1	0.95	[0.93-0.97]	<0.01	0.95	[0.93-0.98]	<0.01	0.99	[0.96-1.0]	0.52
**Dichotomized antibody response according to OD ratio threshold**									
PF13 positivity	0.79	[0.60-1.04]	0.10	0.98	[0.74-1.30]	0.90	1.2	[0.86-1.6]	0.30
MSP1 positivity	0.44	[0.34-0.57]	<0.01	0.44	[0.33-0.57]	<0.001	0.71	[0.53-0.93]	0.01
**Dichotomized antibody response according to MFI Ratio threshold**									
PFf13 positivity	0.83	[0.63-1.1]	0.17	1.01	[0.7-1.4]	0.91	1.08	[081–1.45]	0.57
MSP1 positivity	0.61	[0.47-0.79]	<0.01	0.61	[0.4-0.8]	<0.001	0.89	[0.67-1.18]	0.43
**Dichotomized antibody response according with ROC threshold**									
PF13 positivity	0.70	[0.5-0.9]	0.03	0.89	[0.7-1.2]	0.48	1.1	[0.8-1.5]	0.48
MSP1 positivity	0.78	[0.60-1.02]	0.07	0.82	[0.6-1.1]	0.21	1.05	[0.78-1.4]	0.75

## Discussion

Many sero-epidemiological data in the field of malaria have been obtained using ELISA, including studies investigating the association of antibody responses to one or more antigens with exposure, transmission intensity and/or with protection against clinical malaria [6, 25–34]. This resulted in multiple associations of individual responses with one or other infection outcome endpoint. Interpretation of such studies is unclear, as indeed malaria parasites express a large array of antigens, eliciting a large antibody repertoire that evolves as exposure increases. Multiplex assays in the format of protein microarrays [10–12, 35, 36], Luminex system [13–16, 37] allow high throughput, flexible investigation of multiple antibody specificities in a single assay. The work reported here aimed to be a step bridging ELISA-based antibody monitoring with a multiplexing format. This investigation was focused on two antigens, the merozoite surface antigen MSP1p19 and the variant erythrocyte surface antigen PF13-DBL1a, as responses to these antigens have been assessed using ELISA in previous sero-epidemiological studies conducted in Senegal [6, 18, 24, 25]. It was of interest to know how the results obtained using both methodologies compared for each antigen in order to be able, in future analysis, to integrate - or not - previous ELISA results with future multiplexing data.

A good overall correlation was found between the two techniques. Prevalence values tended to be lower in the MBA format than estimated from ELISA but the age-associated distribution of antibody levels, were similar. Thus, results found here with these two antigens confirm reports from other groups comparing multiplex antibody measurements and traditional monoplex ELISA that showed a high degree of correlation [15, 37, 38]. Further, assessment of antibody responses using the two methods did not lead to concordant conclusions regarding the association of the antibody responses with protection against clinical malaria in the subsequent 5.5-month transmission season. It is important to note that the association of MSP1p19 ELISA responses with protection observed here is consistent with previous findings in this setting conducted in 2000, although in previous studies protection against clinical malaria was also associated with elevated ELISA-based OD ratios (*P* <0.01, RR = 0.66 [0.51-0.86] in an age-adjusted model [6]. Such relationship between elevated ELISA-based IgG responses (dichotomized OD ratios >7) and protection against clinical malaria was not found here, possibly related to the different level of transmission resulting in lower antibody boosting (17.9 infective bites/individual in 2002 *vs* 50.75 in 2000).

The discrepant association of ELISA and MBA readouts with protection is of concern for analysis aiming at combining data from different studies using one or the other methodology. In the present work, it may result from the relatively limited size of the susceptible age groups in the cohort studied, resulting in non-reproducible association with individual antibody-level readouts. It may however reflect an important intrinsic difference, namely that both formats display incompletely overlapping sets of epitopes. This is not unexpected as covalent cross-linking to the beads and adsorption to the plastic wells impact differently on the panel of epitopes displayed. As a consequence, antibody specificities captured do not fully overlap in the two systems, translating into an imperfect correlation of antibody levels measured by both technologies (Rho = 0.74 in the case of MSP1p19). Whether some of protection-associated epitopes of MSP1p19 are exposed in the ELISA assay while masked in the MBA format definitely needs to be further explored. In addition, it is worth recalling that a large body of data indicates that association with protection is more accurately investigated using functional tests, such as growth inhibition assays [39, 40], ADCI [41] or neutrophil respiratory burst [42] than using simple antibody-level measures. It is possible that antibody investigation using individual antigens or multiplex formats is better suited to analysis of antigenicity in human populations and of dynamics of responses in different endemic conditions.

There was no association of any of the PF13 antibody response read out with protection against clinical malaria. This is not surprising as PF13-0003, the variant antigen from which PF13 is derived, is merely one amongst a large array of variant surface antigens that elicit a variant-specific surface seroreactivity [18] and hence probably variant-specific protection. It is thus predicted that antibody to PF13 would be protective against PF13 and closely related variants but not against unrelated variants (*i.e.* the great fraction of the *var* repertoire). To properly define protection afforded by anti PF13 antibody, analysis of the *var* genes expressed by superinfecting parasites is needed to demonstrate that these do not include PF13/PF13-like genes. This could not be done because parasites were not appropriately collected for ribonucleic acid (RNA) extraction. Nevertheless, data are consistent with the current view that antibody to PF13 are acquired by young children at the time they experience numerous infections displaying a large array of antigens, including a wide repertoire of PfEMP1 antigens.

The age distribution of seroprevalence and levels of antibody to MSP1p19 in the 2002 survey are in line with observations made in a previous cross-sectional survey conducted in Ndiop in 2000 showing 79% of responders [6]. With regard to antibodies to PF13, the age distribution in this meso-endemic setting differs from that in the nearby village of Dielmo, where malaria is holo-endemic [18]. In Dielmo, seroprevalence was high (99.1% as assessed by ELISA on PF13) and seroconversion occurred before the age of five years. In contrast, a seroprevalence of 71% (as assessed by ELISA) was observed here in Ndiop, and seroconversion occurred over a much longer time period, namely the first 14 years of life. Thus, there is a clear age shift in the acquisition of antibody to PF13 in Ndiop compared to Dielmo. This is consistent with the differing transmission conditions in both settings, the EIR being five to ten-fold lower in Ndiop than in Dielmo, and transmission being restricted over a few months in Ndiop while being perennial in Dielmo.

Interestingly, a bell-shape distribution of antibody to PF13 by age was observed, with adult individuals having lower antibody levels than older children. This confirms previous observations in Dielmo [18]. Such age-distribution of IgG responses can be interpreted as indicating limited boosting of the PF13 response by infections in older individuals, as i) only a small fraction of each parasite inoculum expresses PF13; and, ii) anti PF13 antibody would readily eliminate PF13- and PF13-related variants. This differs from the continuous increase in antibody levels observed for MSP1-P19 as people get older. In this case, the whole inoculum of the successive parasite infections expresses this well-conserved and abundant antigen, probably triggering frequent boosting.

## Conclusion

This duplex pilot study provided important information for future work. Although there was a strong overall concordance of responses between both methodologies, there was a clear difference when analysing the association with protection, possibly reflecting partial overlap of displayed epitopes. Merging data generated in previous studies using ELISA with future studies using the MBA format needs to be done with some caution. This work indicates that MBA is a robust and cost-effective approach for seroprevalence studies, expression of results for antibody responses can be conveniently done using MFI values and calls for expanding the antigen array in future studies.

## Electronic supplementary material

Additional file 1: **Age distribution of IgG responses to PF13 and PfMSP1p19 expressed as OD for ELISA and MFI for MBA.** OD and MFI values of IgG responses to PF13 and MSP1p19 are plotted as histograms (mean + SE). Antibody responses were stratified according to five age groups (<5, 5–9, 10–14, 15–29 and >30 years; symbols used range from empty, pale grey, light grey, dark grey and black, respectively). Brackets and asterisk indicate significant differences (*P* <0.05) between levels of antibody responses in different age groups. (PDF 50 KB)
