# Supplementary figures and images for: Comparative analysis of IgG responses to Plasmodium falciparum MSP1p19 and PF13-DBL1α1 using ELISA and a magnetic bead-based duplex assay (MAGPIX®-Luminex) in a Senegalese meso-endemic community
Source: Malar J. 2014 Oct 17;13:410. doi: 10.1186/1475-2875-13-410 (PMC4221706; doi:10.1186/1475-2875-13-410)

Age-groups  <5  5-9  10-14  15-29  ≥30

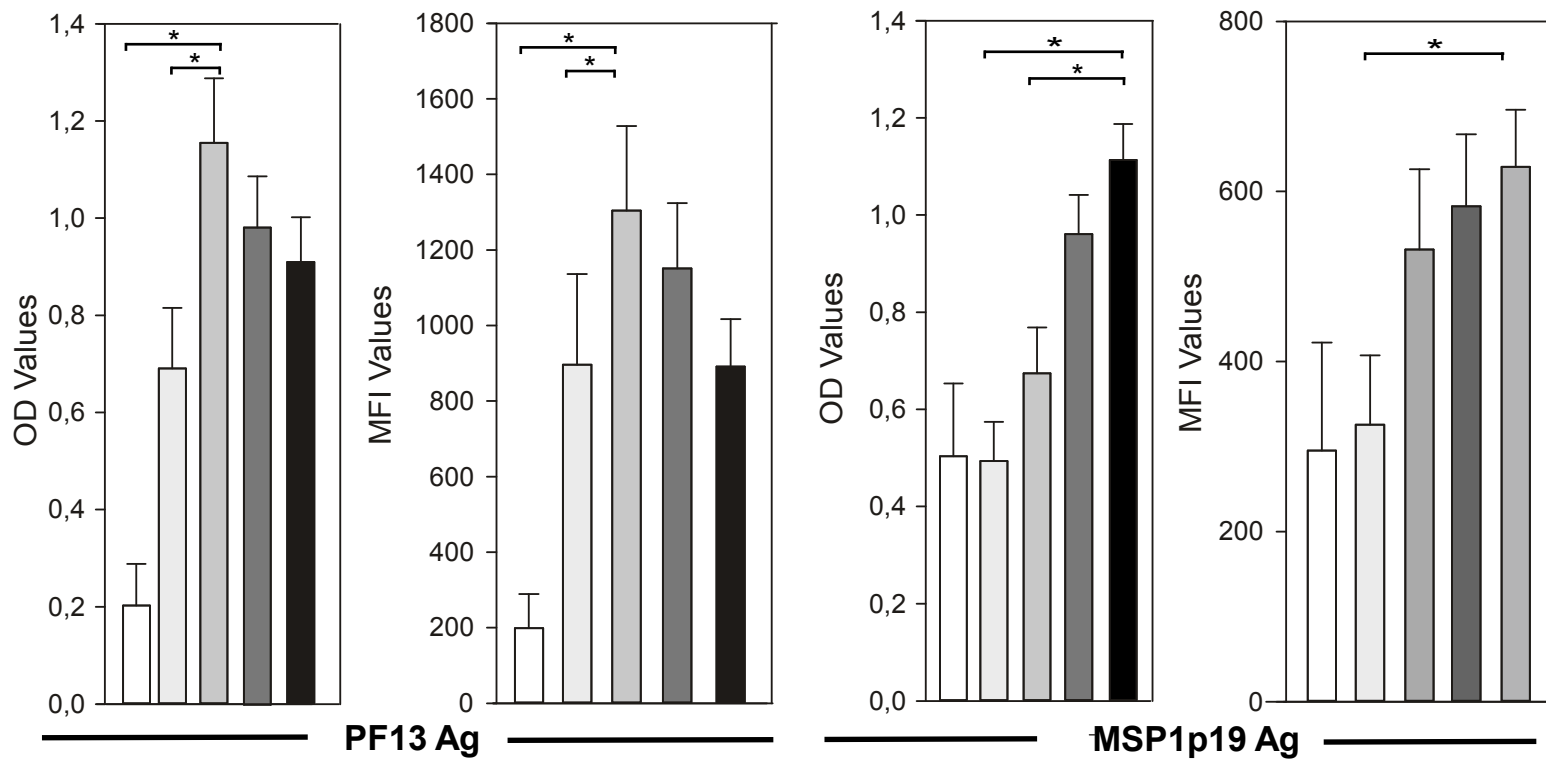

Supplement: Supplementary file 1 — Additional file 1: Age distribution of IgG responses to PF13 and PfMSP1p19 expressed as OD for ELISA and MFI for MBA. OD and MFI values of IgG responses to PF13 and MSP1p19 are plotted as histograms (mean + SE). Antibody responses were stratified according to five age groups (<5, 5–9, 10–14, 15–29 and >30 years; symbols used range from empty, pale grey, light grey, dark grey and black, respectively). Brackets and asterisk indicate significant differences (P <0.05) between levels of antibody responses in different age groups. (PDF 50 KB) [file 12936_2014_3579_MOESM1_ESM.pdf]
